# The effectiveness and safety of computed tomographic peritoneography and video-assisted thoracic surgery for hydrothorax in peritoneal dialysis patients: A retrospective cohort study in Japan

**DOI:** 10.1371/journal.pone.0238602

**Published:** 2020-09-03

**Authors:** Naoya Matsuoka, Makoto Yamaguchi, Akimasa Asai, Keisuke Kamiya, Hiroshi Kinashi, Takayuki Katsuno, Takaaki Kobayashi, Hirofumi Tamai, Takatoshi Morinaga, Takaaki Obayashi, Kichio Nakabayashi, Shigehisa Koide, Michimasa Nakanishi, Katsushi Koyama, Yasuhiro Suzuki, Takuji Ishimoto, Masashi Mizuno, Yasuhiko Ito

**Affiliations:** 1 Department of Nephrology and Rheumatology, Aichi Medical University, Nagakute, Japan; 2 Department of Renal Transplant Surgery, Aichi Medical University, Nagakute, Japan; 3 Department of Nephrology, Anjo-Kosei Hospital, Anjo, Japan; 4 Department of Nephrology, Narita Memorial Hospital, Toyohashi, Japan; 5 Department of Nephrology, Fujita Health University School of Medicine, Toyoake, Japan; 6 Division of Nephrology and Rheumatology, Department of Internal Medicine, Kariya Toyota General Hospital, Kariya, Japan; 7 Department of Nephrology and Renal Replacement Therapy, Nagoya University Graduate School of Medicine, Nagoya, Japan; Taichung Veterans General Hospital, TAIWAN

## Abstract

**Introduction:**

Albeit uncommon, hydrothorax is an important complication of peritoneal dialysis (PD). Due to paucity of evidence for optimal treatment, this study aimed to evaluate the effectiveness and safety of computed tomographic (CT) peritoneography and surgical intervention involving video-assisted thoracic surgery (VATS) for hydrothorax in a retrospective cohort of patients who underwent PD in Japan.

**Methods:**

Of the 982 patients who underwent PD from six centers in Japan between 2007 and 2019, 25 (2.5%) with diagnosed hydrothorax were enrolled in this study. PD withdrawal rates were compared between patients who underwent VATS for diaphragm repair (surgical group) and those who did not (non-surgical group) using the Kaplan-Meier method and log-rank test.

**Results:**

The surgical and non-surgical groups comprised a total of 11 (44%) and 14 (56%) patients, respectively. Following hydrothorax diagnosis by thoracentesis and detection of penetrated sites on the diaphragm using CT peritoneography, VATS was performed at a median time of 31 days (interquartile range [IQR], 20–96 days). During follow-up (median, 26 months; IQR, 10–51 months), 9 (64.3%) and 2 (18.2%) patients in the non-surgical and surgical groups, respectively, withdrew from PD (*P* = 0.021). There were no surgery-related complications or hydrothorax relapse in the surgical group.

**Conclusions:**

This study demonstrated the effectiveness and safety of CT peritoneography and VATS for hydrothorax. This approach may be useful in hydrothorax cases to avoid early drop out of PD and continue PD in the long term. Further studies are warranted to confirm these results.

## Introduction

Albeit uncommon, hydrothorax is an important complication in patients on peritoneal dialysis (PD), because it is one of the causes of patients dropping out of PD early [1–5]. Hydrothorax results from the migration of dialysate from the peritoneal cavity into the pleural space through pleuroperitoneal leakage [1, 2]. Previous studies [1–7] have reported treatment strategies for hydrothorax, including conservative and surgical approaches; nonetheless, the optimal treatment method has not been fully established.

Following a diagnosis of hydrothorax in patients, physicians primarily select the conservative approach as the first step—namely, PD interruption, peritoneal dialysate volume reduction, or shortening the dwell period or avoiding an overnight dwell, making it possible to continue PD. However, the cure rate is, at best, 50% with conservative therapy, ultimately leading to the requirement of a permanent transfer to hemodialysis (HD) [1–7]. Based on these studies, some recent case reports have indicated the effectiveness of a surgical approach involving video-assisted thoracoscopic surgery (VATS) for cases refractory to conservative treatment, resulting in higher success rates [8–14]. However, little is known about the standardized optimal method for surgical intervention. Furthermore, no previous study has evaluated the impact of surgical intervention on continuing PD, compared to conservative methods longitudinally. Hence, this study aimed to assess the effectiveness and safety of a surgical method involving VATS for hydrothorax in combination with a diagnostic tool to detect the penetrated sites on the diaphragm using CT peritoneography and compare it with a non-surgical approach in a large multicenter retrospective PD cohort in Japan.

## Materials and methods

### Statement of ethics

The study protocol and consent procedure were approved by the ethics committees of Aichi Medical University, Nagoya University, Fujita Health University, Kariya General Hospital, Narita Memorial Hospital, and Anjo Kosei Hospital (approval number: 2019–144). The requirement for the acquisition of informed consent from patients was waived owing to the retrospective nature of this study.

### Study population and data source

**Fig 1:**
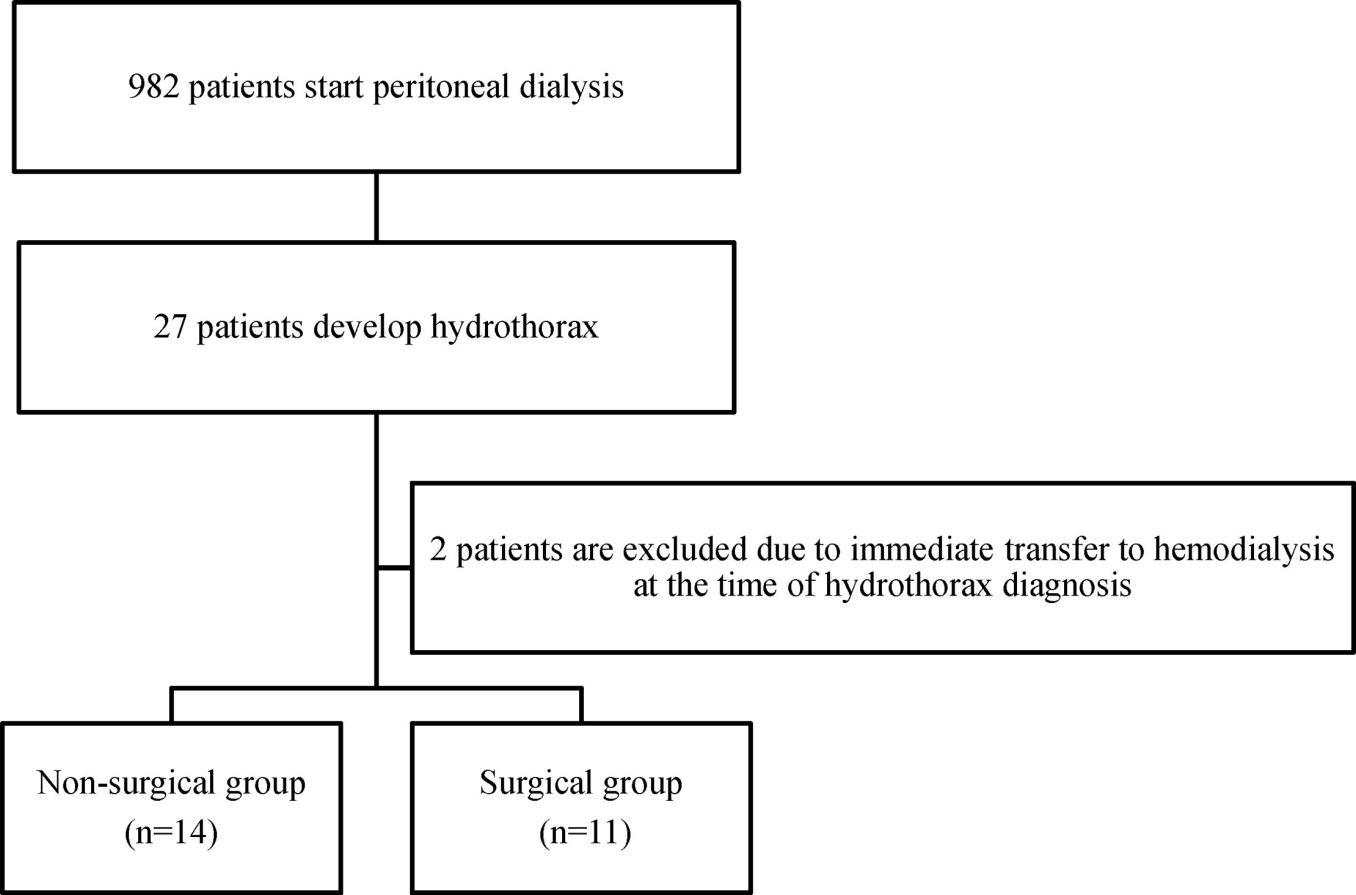
Flow diagram of patient selection.

We conducted a retrospective cohort study that included a total of 982 adult patients aged ≥20 years who underwent PD as renal replacement therapy between January 2007 and December 2019 at the following six nephrology centers in Japan: Aichi Medical University, Nagoya University, Fujita Health University, Kariya General Hospital, Narita Memorial Hospital, and Anjo Kosei Hospital. Of the total patients, 27 (2.7%) developed hydrothorax during the observation period. Of these 27 patients with hydrothorax, we excluded two patients who withdrew from PD at the time of hydrothorax diagnosis: one developed severe bleeding when thoracentesis was performed for diagnosis, and the other underwent a hybrid of HD and PD at the time of hydrothorax diagnosis and was immediately transferred to HD only. Finally, a total of 25 patients were included in the present study and were categorized into two groups, namely, the surgical group, which comprised 11 (44%) patients who were treated using the surgical approach involving VATS, and the non-surgical group, which comprised 14 (56%) patients who did not undergo surgical intervention. Two treatment methods were chosen as per routine clinical care, depending on the physician’s discretion (shown in Fig 1).

All data were fully anonymized (S1 Table).

### Data collection

We collected data on baseline characteristics at the time of hydrothorax diagnosis, including age, sex, cause of kidney disease (diabetic nephropathy, glomerulonephritis, renal sclerosis, polycystic kidney disease, or others), penetrated site (right or left), number of penetrated sites in the diaphragm on computed tomographic (CT) peritoneography, onset time after PD initiation, cause of PD withdrawal (hydrothorax, peritonitis, ultrafiltration failure, impairment in activities of daily living [ADL], or others), clinical symptoms, the weekly Kt/V (kidney and peritoneal) and, in the surgical group, time from the diagnosis of hydrothorax to operation (days). Furthermore, we compared the change in urine output (mL/day) before and after intraperitoneal contrast media injection when performing CT peritoneography and focused on PD duration until withdrawal. PD withdrawal was defined as transferring to HD. Patients were followed up until PD withdrawal or other censoring events, including death, or until the end of follow-up for the present study, whichever occurred first. Patients and their caregivers underwent a standard training program after catheterization.

### Diagnostic and treatment approach

As the first step, we evaluated pleural effusion by thoracentesis with pleural fluid analysis (a higher glucose concentration in the pleural fluid than in the plasma, as previously reported [1]) to find the presence of hydrothorax. In the second step, we used CT peritoneography as the diagnostic modality for detecting the penetrated sites on the diaphragm; in the case of surgical intervention, VATS was performed subsequently.

### CT peritoneography evaluation

CT peritoneography was performed using dialysate mixed with iodinated contrast. The dose of the iodinated contrast used was approximately 1 mL/kg per 30 mL/kg of dialysate, as previously reported [15]. After 30 minutes in the supine position, CT was performed.

### Operation method for video-assisted thoracic surgery

Under general anesthesia, the patient was intubated using a double-lumen endotracheal tube and was placed in a hemilateral decubitus position. After instilling indigo carmine into the peritoneal cavity, as previously reported [11, 12], making it possible to identify a visible repairable communication, the penetrated sites were closed.

### Statistical analysis

Differences in clinical characteristics and outcomes were compared between the surgical and non-surgical groups using the Wilcoxon rank-sum test or Fisher’s exact test. The Wilcoxon rank-sum test was performed on the continuous variables to assess the significance of intergroup differences, while Fisher’s exact test was employed to compare the categorical variables, which are expressed as percentages. A paired *t*-test was used to compare urine volume before CT peritoneography and at 1 week after the examination. The cumulative probability of PD withdrawal was calculated using the Kaplan-Meier method and the log-rank test. All statistical analyses were performed using JMP version 14.0.0 (SAS Institute, Cary, NC, USA), and the statistical significance level was set at *P* <0.05.

## Results

### Study participants and clinical characteristics of the groups at baseline

The clinical characteristics of the surgical and non-surgical groups are summarized in Table 1. In both groups, presenting symptoms and signs of pleural effusion included dyspnea and inadequate ultrafiltration ability, and most cases presented with right-sided pleural effusion. The diagnosed timing of hydrothorax after PD initiation was earlier in the surgical group than in the non-surgical group (median 1.5 months [IQR, 0.5–32.4 months] vs. median 8.6 months [IQR, 0.9–11.1 months], respectively), although there were no significant differences (P = 0.511). In the surgical group, operations were performed at a median of 31 days (IQR, 20–96 days) following a diagnosis of hydrothorax. The other factors at baseline were not significantly different between the two groups.

**Table 1:** Comparison of clinical characteristics between surgical (*n* = 11) and non-surgical (*n* = 14) groups.

	Non-surgical group (*n* = 14)	Surgical group (*n* = 11)	*P-*value
**Baseline characteristics**			
Age (year)	63 (59–68)	55 (43–71)	0.207
Male (*N* (%))	11 (78.6)	6 (54.6)	0.201
Cause of kidney disease (*N* (%))			0.488
Diabetic nephropathy	7 (50.0)	3 (27.3)	
Glomerulonephritis	1 (7.1)	4 (36.4)	
Renal sclerosis	1 (7.1)	1 (9.1)	
Polycystic kidney disease	2 (14.3)	2 (18.2)	
Others	3 (21.4)	1 (9.1)	
Onset time after PD initiation (months)	8.6 (0.9–11.1)	1.5 (0.5–32.4)	0.511
Clinical symptoms (*N* (%))			0.610
Dyspnea	9 (64.3)	9 (81.8)	
Inadequate ultrafiltration	3 (21.4)	1 (9.1)	
Asymptomatic	2 (14.3)	1 (9.1)	
Right side affected (*N* (%))	14 (100)	10 (90.9)	0.250
Number of penetrated sites in the diaphragm on CT peritoneography	1 (1–1)	1 (1–1)	0.357
Time from the diagnosis of hydrothorax to operation (days)		31 (20–96)	
Dose of dialysis			
Total Kt/V	1.7 (1.5–2.1)	1.8 (1.5–2.5)	0.597
Kidney Kt/V	1.2 (0.6–1.3)	0.8 (0.6–0.8)	0.113
Peritoneal Kt/V	0.9 (0.5–1.0)	1.2 (0.6–1.9)	0.105
**Outcomes**			
PD withdrawal (*N* (%))	9 (64.3)	2 (18.2)	0.021*
Cause of PD withdrawal (*N* (%))			
Hydrothorax	8 (88.9)	0 (0)	
Peritonitis	0 (0)	0 (0)	
Ultrafiltration failure	0 (0)	1 (50.0)	
Impairment in ADL	1 (11.1)	1 (50.0)	
Observation period (months)	5.7 (0.2–43.4)	18.3 (6.7–40.1)	0.228

Continuous data are presented as a median (interquartile range), and categorical data are expressed as a number (proportion).

*P-value <0.05 is considered significant.

Abbreviations: ADL, activities of daily living; eGFR, estimated glomerular filtration rate; PD, peritoneal dialysis; CT, computed tomographic.

### Findings of CT peritoneography

**Fig 2:**
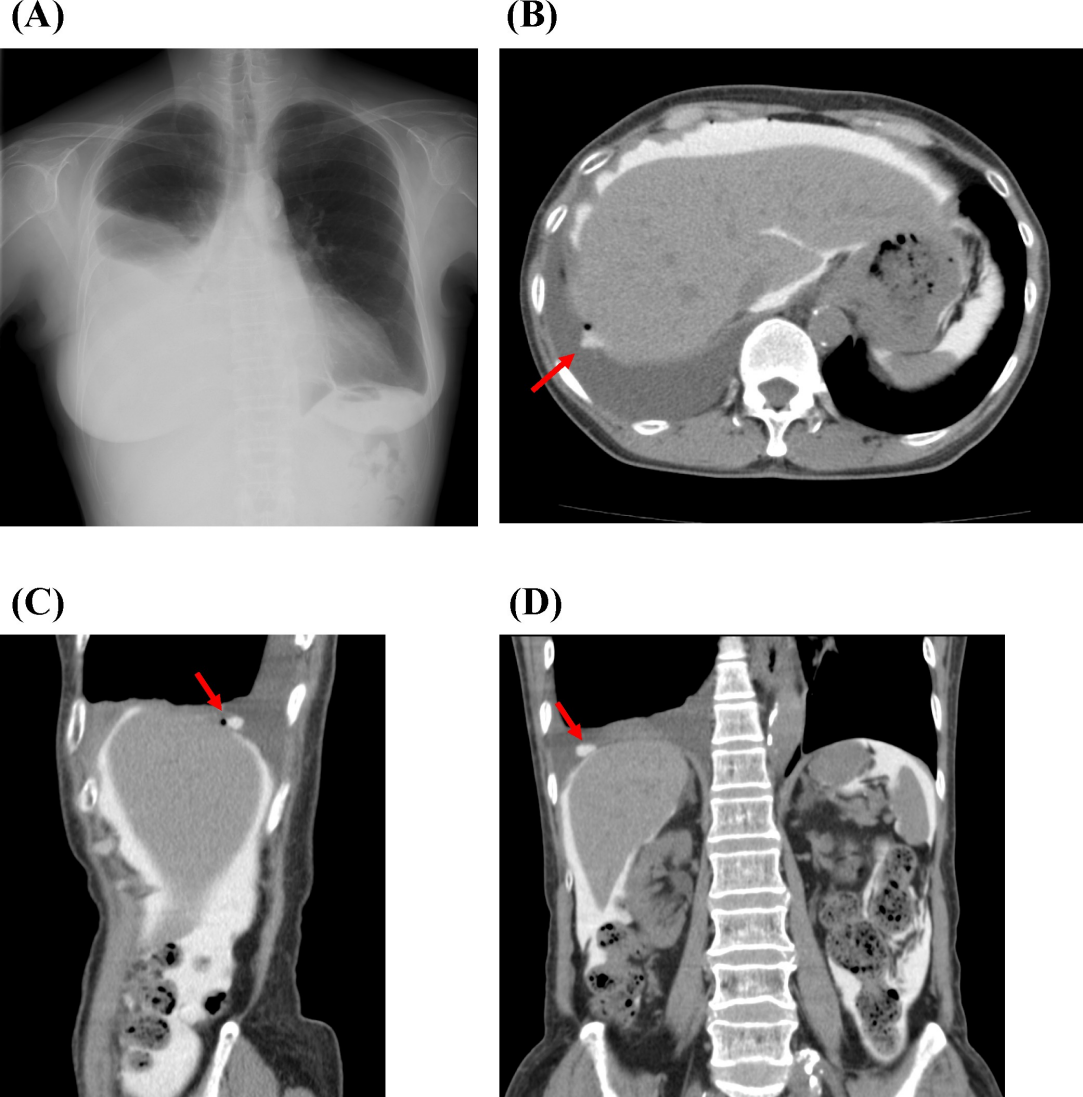
A case of hydrothorax in a peritoneal dialysis patient with a penetrated site. (A) Chest x-ray at the time of hydrothorax diagnosis. (B, C, D) Penetrated sites on the diaphragm as revealed by CT peritoneography on the transverse, sagittal, and coronal view, respectively (red arrows are penetrated sites).

**Fig 3:**
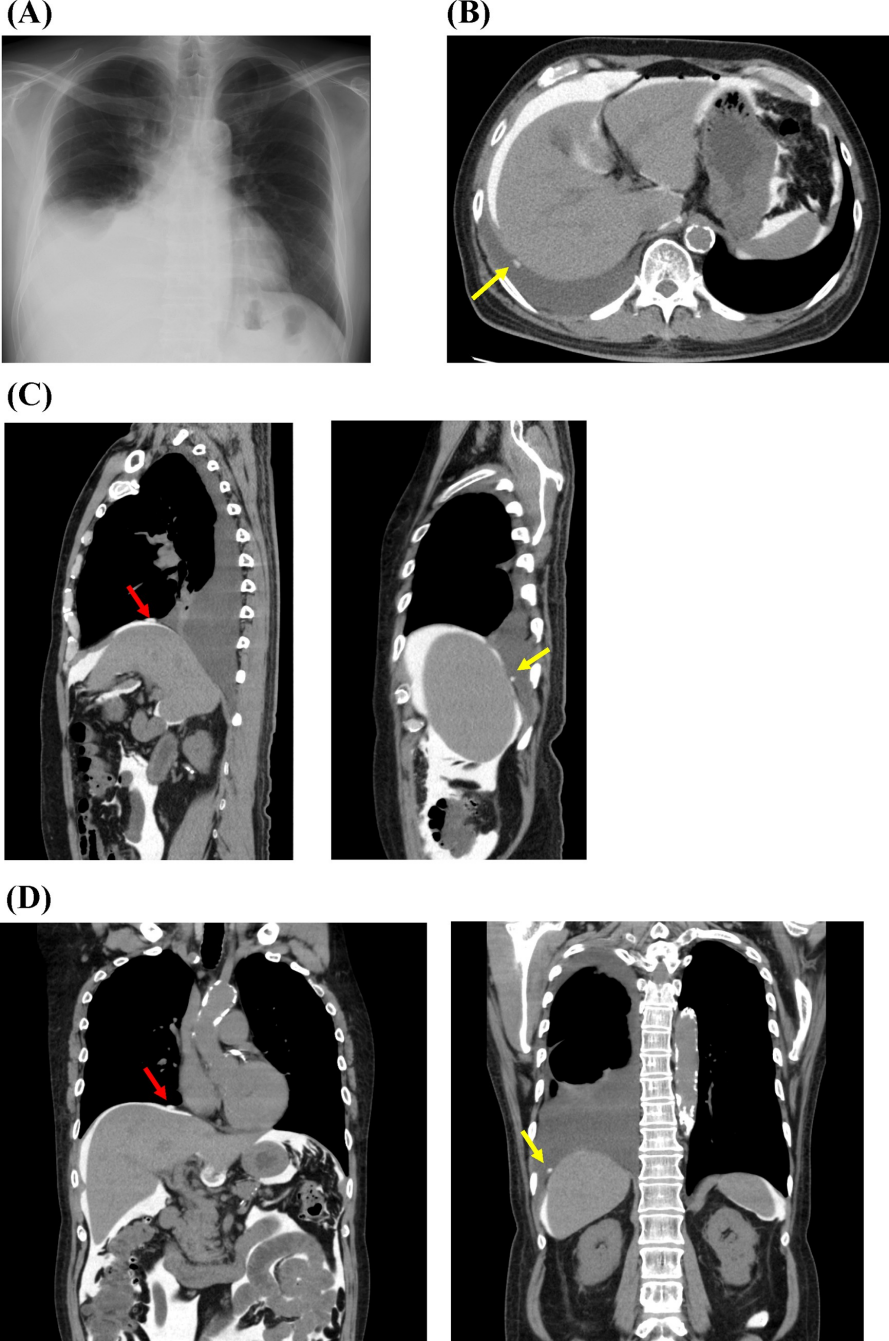
A case of hydrothorax in a peritoneal dialysis patient with two possible penetrated sites. (A) Chest x-ray at the time of hydrothorax diagnosis. (B, C, D) Penetrated sites on the diaphragm as revealed by CT peritoneography on the transverse, transverse, sagittal, and coronal view, respectively (red and yellow arrows are penetrated sites).

CT peritoneography could reveal the penetrated sites on the diaphragm, and in some cases, multiple defects on the diaphragm (shown in Figs 2 and 3). All patients who underwent CT peritoneography did not exhibit any change in urine volume before and at 1 week after the examination (urine volume before examination: 1200 [IQR, 1200–1350] mL/day; urine volume after examination: 1300 [IQR, 816–1500] mL/day; *P* = 0.671) (Table 2), suggesting that CT peritoneography had no considerable effect on residual kidney function.

**Table 2:** Urine volume before and 1 week after CT peritoneography (n = 25).

	Before CT peritoneography	After CT peritoneography	*P-*value
Urine volme (ml/day)	1200 (1200–1350)	1300 (816–1500)	0.671

Continuous data are presented as medians (interquartile range).

Abbreviations: CT, computed tomographic.

### Procedure of VATS

**Fig 4:**
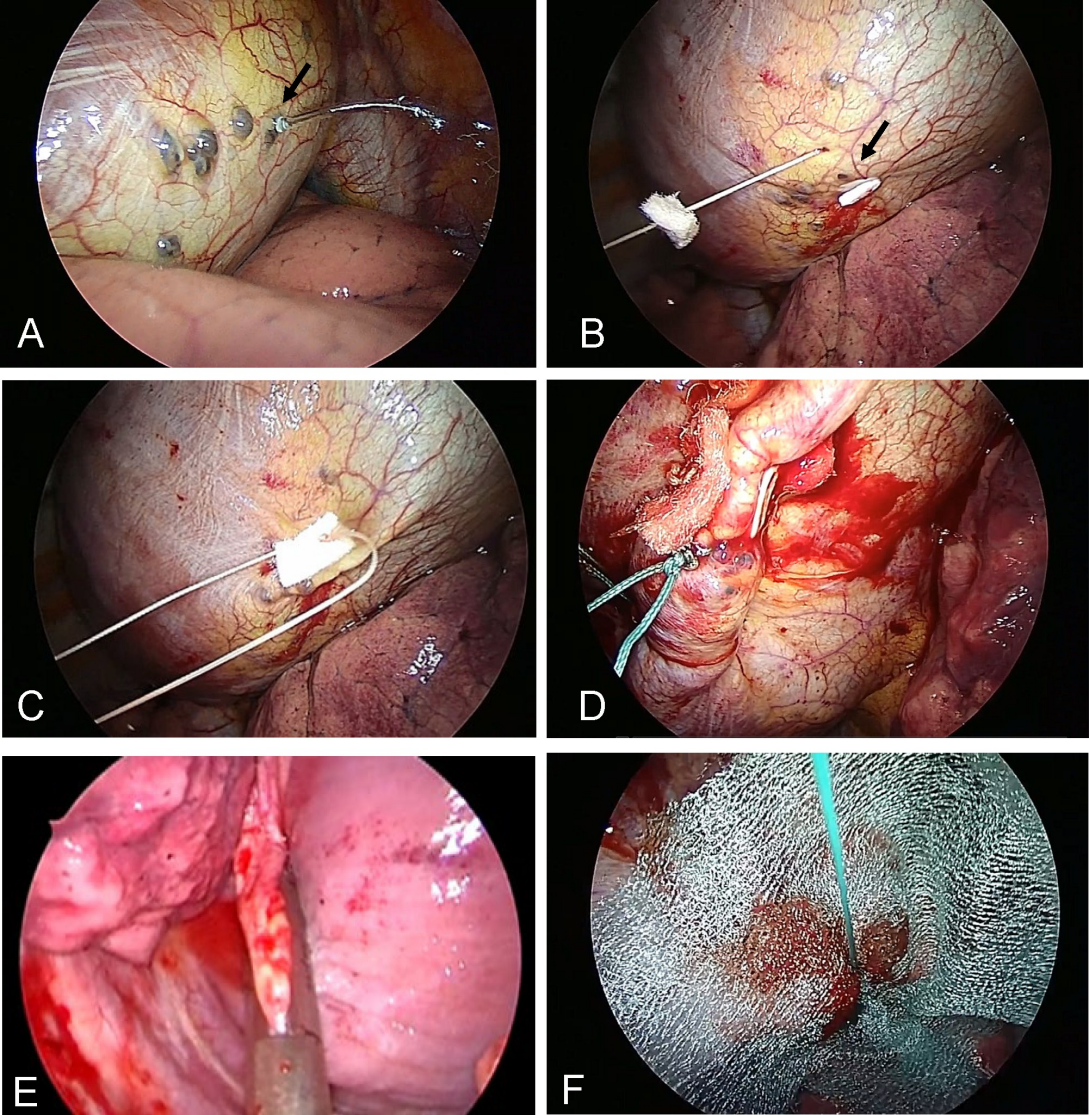
Procedure of VATS. **(A)** Multiple blebs with blue staining were identified on the diaphragm. Dialysis solution was flowed through a small pore (arrow). **(B–D)** Penetrated sites on the diaphragm (arrow) were closed by direct suturing. (**E**) Penetrated sites on the diaphragm were closed by surgical stapler. (**F**) The absorbable polyglycolic acid sheet covering to reinforce the diaphragm.

During the operation, based on the defective sites on the diaphragm as revealed by CT peritoneography, we identified a repairable communication site by instilling 20 mg of indigo carmine with 2 liters of PD dialysate into the peritoneal cavity (shown in Fig 4A, and S1 Video) [11, 12]. Subsequently, we inspected the diaphragm using a thoracoscope, and the lesion was closed by direct suturing using 3–0 absorbable multifilament suture (shown in Fig 4B, 4C and 4D, and S1 Video) or an Endo-GIA surgical stapler (shown in Fig 4E). The central tendon around the reinforcement was covered with an absorbable polyglycolic acid sheet (Neoveil®; Gunze, Osaka, Japan) (shown in Fig 4F and S1 Video).

### Outcome data

**Fig 5:**
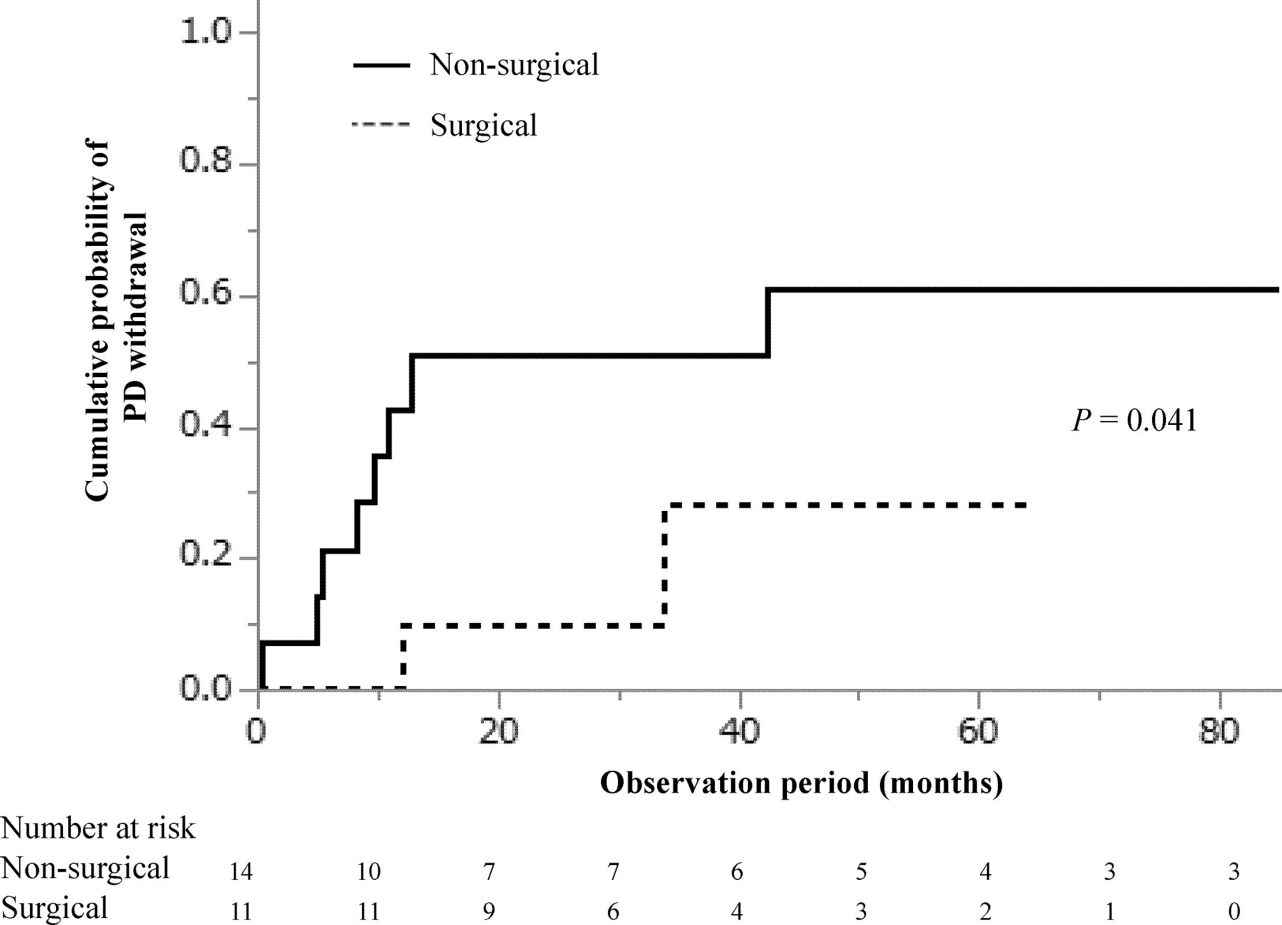
Cumulative probability of withdrawal from peritoneal dialysis.

During follow-up (median, 26 months; IQR, 10–51 months), PD withdrawal occurred in 9 (64.3%) patients in the non-surgical group and 2 (18.2%) in the surgical group (*P* = 0.021). Of these 11 patients, 1 (11.1%) in the non-surgical group and 1 (50%) in the surgical group received a combined PD and HD therapy before permanently transferring to HD. Concerning the cause of PD withdrawal, 8 (88.9%) patients in the non-surgical group and none (0%) in the surgical group had hydrothorax, 1 (11.1%) patient in the non-surgical group and none (0%) in the surgical group experienced an impairment in ADL because of intracerebral bleeding, no patient had peritonitis in both groups, and in the non-surgical and surgical groups, 0 (0%) and 1 (50%) patient, respectively, had ultrafiltration failure. The cumulative probabilities of PD withdrawal at 1, 3, and 5 years were 0.43, 0.51, and 0.61, respectively, for the conservative group, and 0.0, 0.28, and 0.28, respectively, for the surgical group, indicating that the surgical group had a lower risk of PD withdrawal than the non-surgical group (log-rank test: *P* = 0.040; shown in Fig 5).

In the non-surgical group, only one (1.4%) patient in our study was cured spontaneously.

Meanwhile, no major complications were observed in the surgical group, and the cure rate was 100%. Postoperatively, PD was reinstituted within 1 week without volume restriction of the PD dialysate, and no relapse occurred. The length of hospitalization due to surgery was a median of 12 days (IQR, 5–17 days).

## Discussion

This retrospective cohort study showed that CT peritoneography and surgical intervention involving VATS is an effective and safe treatment strategy for hydrothorax. This study has the following strengths: First, the present study constituted a large PD cohort and had a long observation period (median, 26 months; IQR, 10–51 months). Second, this is the first study to precisely compare the clinical impact of surgical intervention for hydrothorax with that of conservative treatment.

Concerning the modality for detecting the defective sites on the diaphragm after diagnosis of hydrothorax by thoracentesis, previous studies have shown that CT peritoneography could diagnose small leaks, adhesions, loculated fluid collections, intra-abdominal abscesses, and pseudocysts with high sensitivity, specificity, and convenience [15–17]. In the present study, we also used CT peritoneography as the diagnostic modality for detecting the penetrated sites on the diaphragm, following the diagnosis of hydrothorax by thoracentesis. Consequently, we could detect peritoneal defects in all cases, and some cases of multiple penetrated sites, thereby providing useful information for the subsequent surgery. Furthermore, although a previous study has indicated the risk of nephrotoxicity secondary to CT peritoneography [11], no considerable effect on residual kidney function was detected after CT peritoneography in the present study, such as reported in a rat model [18]. These results suggest that CT peritoneography is useful for early and accurate diagnosis of the site of pleural leakage lesion on the diaphragm.

As for the therapeutic intervention, both, non-surgical and surgical methods have been reported. As the first-line approach, conservative methods have usually been chosen, that include peritoneal dialysate volume reduction, shortening the dwell period or avoiding an overnight dwell, or temporary cessation of continuous ambulatory PD. Previous studies have reported that some pleuroperitoneal leaks resolve spontaneously [1–6] by the following presumed mechanism: PD dialysate itself may act as a sclerosant by promoting “sealing” of the epithelial layers, in addition to reducing the pressure gradient between the pleural and peritoneal cavity. However, similar to the present study, only the above conservative approach could not avoid early drop out from PD [1–4].

Concerning other non-surgical treatment methods, chemical pleurodesis may be offered to patients with recurrent pleural effusion who are unresponsive to conservative interventions and need or desire to continue PD. Only a few reports have described the effectiveness of such treatment, and the cure rate was only 67% [19, 20]; therefore, we did not select it for the present cohort.

Concerning the surgical intervention, several case reports have indicated that VATS could be a useful approach to more precisely observe the thoracic cavity [8–14]. However, no standardized method has been established.

In the present study, after detecting the penetrated sites on the diaphragm by CT peritoneography, during operation, instilling indigo carmine in the peritoneal dialysate could help detect the fragile site of the diaphragm visually, leading to the closure of the site by direct suturing or surgical stapler and covering PGA sheet easily. By these surgical interventions in the early phase after diagnosis of hydrothorax, no major complication and no pleural effusion relapse were observed, and a sufficient amount of volume exchange in PD could be obtained early after operation. Therefore, we consider that VATS is indicated for cases in the early phase after diagnosis of hydrothorax, to avoid early drop out of PD.

Based on the results in the present study, we consider that the following diagnostic and treatment strategy for hydrothorax might be effective and safe; a first step of diagnosing hydrothorax by thoracentesis, a second step of detecting penetrated sites on the diaphragm by performing CT peritoneography, and a final step of complete cure by VATS.

This study had some limitations. First, the retrospective nature of the present study introduced bias in decision-making regarding a surgical or non-surgical approach. Second, the treatment method of VATS in the present study is not fully standardized worldwide; therefore, these results should be validated in other large cohort studies.

In conclusion, the present study demonstrated the effectiveness and safety of CT peritoneography and surgical intervention by VATS for hydrothorax. This approach may be useful in the early phase after diagnoses with hydrothorax to continue PD in the long term. Further studies are warranted to confirm these results.

## Supporting information

S1 Table The anonymous data set of the present study. (XLSX)

S1 Video Operation method for hydrothorax. (MP4)

## References

[1] Lew SQ. Hydrothorax: pleural effusion associated with peritoneal dialysis. Perit Dial Int. 2010;30: 13–18. 10.3747/pdi.2008.00168 20056973

[2] Gagnon RF, Daniels E. The persisting pneumatoenteric recess and the infracardiac bursa: possible role in the pathogenesis of right hydrothorax complicating peritoneal dialysis. Adv Perit Dial. 2004;20: 132–136. 15384813

[3] Nomoto Y, Suga T, Nakajima K, Sakai H, Osawa G, Ota K, et al Acute hydrothorax in continuous ambulatory peritoneal dialysis–a collaborative study of 161 centers. Am J Nephrol. 1989;9: 363–367. 10.1159/000167997 2679094

[4] Garcia Ramón R, Carrasco AM. Hydrothorax in peritoneal dialysis. Perit Dial Int. 1998;18: 5–10. 9527025

[5] Abraham G, Shokker A, Blake P, Oreopoulos OG. Massive hydrothorax in patients on peritoneal dialysis: a literature review. Adv Perit Dial. 1988;4: 121–125.

[6] Simmons LE, RaufMir A. A review of management of pleuroperitoneal communication in five CAPD Patients. Adv Perit Dial. 1989;5: 81–83. 2577434

[7] Szeto CC, Chow KM. Pathogenesis and management of hydrothorax complicating peritoneal dialysis. Curr Opin Pulm Med. 2004;10: 315–319. 10.1097/01.mcp.0000127901.60693.d0 15220759

[8] Pattison CW, Rodger RS, Adu D, Michael J, Matthews HR. Surgical treatment of hydrothorax complicating continuous ambulatory peritoneal dialysis. Clin Nephrol. 1984;21: 191–193. 6705282

[9] Saito M, Nakagawa T, Tokunaga Y, Kondo T. Thoracoscopic surgical treatment for pleuroperitoneal communication. Interact Cardiovasc Thorac Surg. 2012;15: 788–789. 10.1093/icvts/ivs193 22753435 PMC3445346

[10] Mitsuboshi S, Maeda H, Kanzaki M. Video-assisted thoracic surgery for pleuroperitoneal communication. Surg Case Rep. 2019;5: 34 10.1186/s40792-019-0595-8 30783792 PMC6381193

[11] Tang S, Chui WH, Tang AW, Li FK, Chau WS, Ho YW, et al Video‐assisted thoracoscopic talc pleurodesis is effective for maintenance of peritoneal dialysis in acute hydrothorax complicating peritoneal dialysis. Nephrol Dial Transplant. 2003;18: 804–808. 10.1093/ndt/gfg042 12637652

[12] Chow KM, Szeto CC, Li PK. Management options for hydrothorax complicating peritoneal dialysis. Semin Dial. 2003;16: 389–394. 10.1046/j.1525-139x.2003.16080.x 12969393

[13] Ratajczak A, Lange-Ratajczak M, Bobkiewicz A, Studniarek A. Surgical management of complications with peritoneal dialysis. Semin Dial. 2017;30: 63–68. 10.1111/sdi.12538 27596540

[14] Sato S, Koike T, Hashimoto T, Tsuchida M. Detection of the communication site by dye injection method at the surgery for pleuroperitoneal communication. Kyobu Geka. 2014;67: 967–970. 25292372

[15] Hollett MD, Marn CS, Ellis JH, Francis IR, Swartz RD. Complications of continuous ambulatory peritoneal dialysis: evaluation with CT peritoneography. Am J Roentgenol. 1992;159: 983–989. 1344976 10.2214/ajr.159.5.1344976

[16] Lam MF, Lo WK, Chu FSK, Fu-Keung L, Yip TPS, Kai-Chung T, et al Retroperitoneal leakage as a cause of ultrafiltration failure. Perit Dial. 2004;24: 466–470. 15490987

[17] Markić D, Zivcić-Cosić S, Valencić M, Miletić D, Rahelić D, Krpina K, et al The role of CT peritoneography as diagnostic tool in patient on peritoneal dialysis with dialysate leakage. Acta Med Croatica. 2011;65: 95–8. 23120824

[18] Bai J, Dong J, Shu J, Xu Y, Ding W, Chen J. Experimental studies on computed tomographic peritoneography. Peritoneal and residual renal function tolerance to iodinated contrast media injected into the peritoneal cavity. Semin Dial. 2020;33: 163–169. 10.1111/sdi.12867 32163640

[19] Sudduth CD, Sahn SA. Pleurodesis for nonmalignant pleural effusions. Recommendations. Chest. 1992;102: 1855–1860. 10.1378/chest.102.6.1855 1446502

[20] Kanaan N, Pieters T, Jamar F, Goffin E. Hydrothorax complicating continuous ambulatory peritoneal dialysis: successful management with talc pleurodesis under thoracoscopy. Nephrol Dial Transplant. 1999;14: 1590–1592. 10.1093/ndt/14.6.1590 10383035

